# Accuracy of generative deep learning model for macular anatomy prediction from optical coherence tomography images in macular hole surgery

**DOI:** 10.1038/s41598-024-57562-5

**Published:** 2024-03-22

**Authors:** Han Jo Kwon, Jun Heo, Su Hwan Park, Sung Who Park, Iksoo Byon

**Affiliations:** 1 Department of Ophthalmology, Biomedical Research Institute, Pusan National University Hospital, Pusan National University School of Medicine, Gudeok-ro 179, Seo-gu, Busan, 49241 South Korea; 2 Department of Ophthalmology, Research Institute for Convergence of Biomedical Science and Technology, Pusan National University Yangsan Hospital, Geumo-ro 20, Mulgeum-eup, Yangsan-si, Gyeongsangnam-do 50612 South Korea

**Keywords:** Deep learning, Generative deep learning model, Macular hole, Optical coherence tomography, Variational autoencoder, Retinal diseases, Data processing, Predictive markers

## Abstract

This study aims to propose a generative deep learning model (GDLM) based on a variational autoencoder that predicts macular optical coherence tomography (OCT) images following full-thickness macular hole (FTMH) surgery and evaluate its clinical accuracy. Preoperative and 6-month postoperative swept-source OCT data were collected from 150 patients with successfully closed FTMH using 6 × 6 mm^2^ macular volume scan datasets. Randomly selected and augmented 120,000 training and 5000 validation pairs of OCT images were used to train the GDLM. We assessed the accuracy and F1 score of concordance for neurosensory retinal areas, performed Bland–Altman analysis of foveolar height (FH) and mean foveal thickness (MFT), and predicted postoperative external limiting membrane (ELM) and ellipsoid zone (EZ) restoration accuracy between artificial intelligence (AI)-OCT and ground truth (GT)-OCT images. Accuracy and F1 scores were 94.7% and 0.891, respectively. Average FH (228.2 vs. 233.4 μm, *P* = 0.587) and MFT (271.4 vs. 273.3 μm, *P* = 0.819) were similar between AI- and GT-OCT images, within 30.0% differences of 95% limits of agreement. ELM and EZ recovery prediction accuracy was 88.0% and 92.0%, respectively. The proposed GDLM accurately predicted macular OCT images following FTMH surgery, aiding patient and surgeon understanding of postoperative macular features.

## Introduction

Full-thickness macular hole (FTMH) is a retinal condition of tractional disruption to the whole foveola, including the Müller cell cone and external limiting membrane (ELM)^1,2^. Idiopathic FTMH is caused by traction to the fovea during posterior hyaloid detachment. Vitrectomy, internal limiting membrane (ILM) peeling, and fluid-air exchange with tamponade can close the macular hole (MH)^3^. For larger holes, additional procedures for ILM can be performed to increase the closure rate^4,5^. Recently, the closure rate of idiopathic FTMH has reached as high as 96–98% after primary vitrectomy with ILM peeling^6–8^. Even if the first ILM flap technique fails to close large holes, the closure rate can reach 100% through reoperation^9^. With ongoing efforts of retinal surgeons for hole closure, most idiopathic FTMH cases can be closed. Therefore, the focus of FTMH surgery has shifted from predicting the hole closure to understanding how macular holes close morphologically and their association with visual outcomes^10^.

Visual acuity (VA) in patients with FTMH is primarily associated with the outer retinal conditions, particularly the ELM and ellipsoid zone (EZ) status. Following a successful closure through vitrectomy, the ELM and EZ show gradual recovery on optical coherence tomography (OCT), leading to improved VA^11,12^. Various factors extractable from preoperative OCT influence ELM and EZ recovery. Lee et al. revealed that axial length, minimum linear diameter, and preoperative intraretinal layer thickness are related to EZ recovery^13^. However, measuring these diverse factors in clinics manually and inputting them into a prediction model may be impractical due to the substantial time and effort.

Deep learning (DL) models have been proposed to identify risk factors or assess retinal disease prognoses from OCT images^14^. Several DL models have been proposed for the prognosis of FTMH surgery. These models utilize a combination of preoperative OCT and clinical data to predict the postoperative hole closure^15^, as well as preoperative OCT to estimate postoperative VA^16^. However, previous DL models for FTMH could not predict the detailed anatomical conditions of the macula, including the ELM and EZ restoration.

Generative deep learning models (GDLMs) may be suitable for constructing expected postoperative OCT images based on preoperative OCT images in eyes with FTMH. GDLMs are divided into the following categories: variational autoencoders (VAEs) and generative adversarial networks (GANs)^17^. VAEs are widely utilized for image-to-image conversion and super-resolution and have shown remarkable outcomes in producing high-quality images in computer vision and art^17^. Because GANs are known to have unstable training and are prone to spatial deformity and mode collapse phenomenon^18^, we selected VAE as the preferred GDLM for a reliable prediction of the postsurgical macular anatomy^19^.

We herein proposed a GDLM based on VAE to predict postoperative OCT images using preoperative OCT images following FTMH surgery. Further, we evaluated the accuracy and performance of the GDLM by comparing the predicted and actual postoperative OCT images, including concordance for neurosensory retinal areas and foveal anatomy, as well as ELM and EZ restoration.

## Methods

### Patient selection and ethics statements

We consecutively enrolled patients with FTMH who underwent pars plana vitrectomy, ILM peeling, and fluid-air exchange between January 2018 and December 2022. The selection of tamponade materials (air or SF_6_ 18%), ILM staining dyes (0.025% brilliant blue G or 0.05% indocyanine green), and additional ILM procedures (inverted ILM flap or autologous ILM insertion) were left to the surgeon’s discretion. The inclusion criteria were FTMH cases identified through macular volume scans using a swept-source OCT device, the DRI OCT-1 Atlantis (Topcon Corp., Tokyo, Japan), before and 6 months after surgery. The eyes were scanned using a 6 × 6 mm^2^ scan protocol centered on the fovea by an experienced technician. The study protocol was approved by the institutional review boards of the Pusan National University Hospital (PNUH, approval no. 2304-016-126) and Pusan National University Yangsan Hospital (PNUYH, approval no. 05-2023-112). One hundred twenty-five patients who had met the inclusion criteria were excluded from the study based on the exclusion criteria summarized in Supplementary Table S1.

### Data collection

Baseline parameters (age, sex, laterality, axial length, best corrected visual acuity [BCVA], central subfield macular thickness [CSMT], hole size, and FTMH stage based on the Gass classification^20^), intraoperative parameters (combined phacoemulsification, ILM peeled area in disc diameter, ILM manipulation technique, surgeon, ILM staining dye, and tamponade material), and 6-month postoperative parameters (BCVA, CSMT, ELM restoration, and EZ recovery) were assessed. The hole size was measured using the ImageNet 6 ver. 1.24 software (Topcon Corp., Tokyo, Japan) by determining the longest distance between the split ends of the ELM^21^. Successful ELM restoration was defined as a continuous ELM line that was clearly distinguishable between the outer nuclear layer and photoreceptor (PRL) in the fovea. Successful EZ recovery defines the continuous bright band between ELM and RPE in postoperative OCT.

### Automatic registration and preparation of macular volume scan datasets

**Figure 1:**
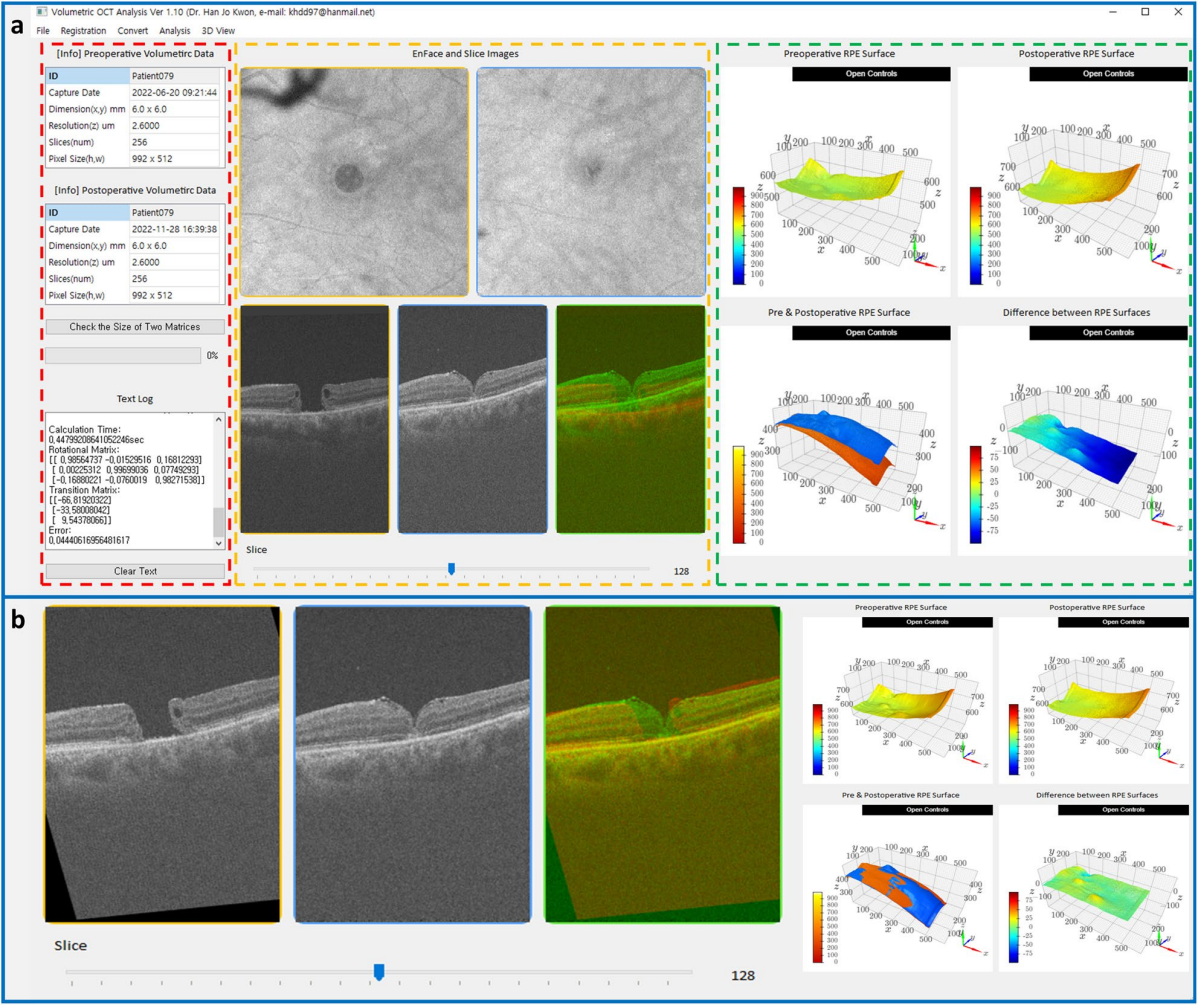
Customized software for automated registration of macular volume scan optical coherence tomography data obtained before and after surgery. To adjust the misalignment between the preoperative and postoperative optical coherence tomography (OCT) data, a custom software was developed using Python (version 3.9.16, Python Software Foundation, Wilmington, Delaware, US) to automatically register the preoperative volumetric data based on the postoperative retinal pigment epithelium (RPE) surface using the least-squares fitting method. (**a**) The registration program comprises three compartments: the left section (red dashed rectangle) contains information from OCT, middle section (yellow dashed rectangle) showcases en face and horizontal cross-sectional OCT images, and right section (green dashed rectangle) exhibits the point clouds of the RPE layer. The lower row of the middle section displays preoperative, postoperative, and merged OCT images. The merged images show the preoperative and postoperative OCT scans in red and green, respectively. Before registration, an inconsistency existed in the RPE layer level between the two OCT images. The right panel shows the preoperative (upper left) and postoperative (upper right) point-cloud sets of the RPE layers. The lower-left image simultaneously shows two point-cloud sets, where the orange and blue clouds represent the preoperative and postoperative RPE layers, respectively. Before the registration, the postoperative RPE level is slightly higher than the preoperative RPE level in the temporal scan space. The difference between the two sets is further illustrated by the pixel differences in the lower-right image. A color scale bar indicating the pixel units is also included in the lower-left corner of each cloud image. (**b**) The customized software illustrates the results of automated registration. A geometric transformation is applied to the preoperative OCT volume scan matrix to ensure that the RPE surfaces in both OCT images are closely aligned. Thus, the preoperative OCT image is rotated and shifted. The right section displays the RPE levels of the two OCT datasets, which are almost indistinguishable. After the registration, the error between the two RPE layers approached zero.

The macular volume OCT scan contains 256 continuous slices, each consisting of 992 vertical and 512 horizontal pixels of an 8-bit grayscale image, stored in a three-dimensional (3D) array using the code distributed by Graham (Graham, M. 2020. OCT-Converter. Version v0.5.0. https://github.com/marksgraham/OCT-Converter. Accessed 7 March 2023). It is evident that the retinal vasculature and retina itself can change after FTMH surgery^22^. Choroid may also vary post-vitrectomy in patients with vitreous traction^23^. Hence, we based our registration on the RPE surface, which was expected to undergo minimal changes and was not manipulated during surgery (Fig. 1).

**Figure 2:**
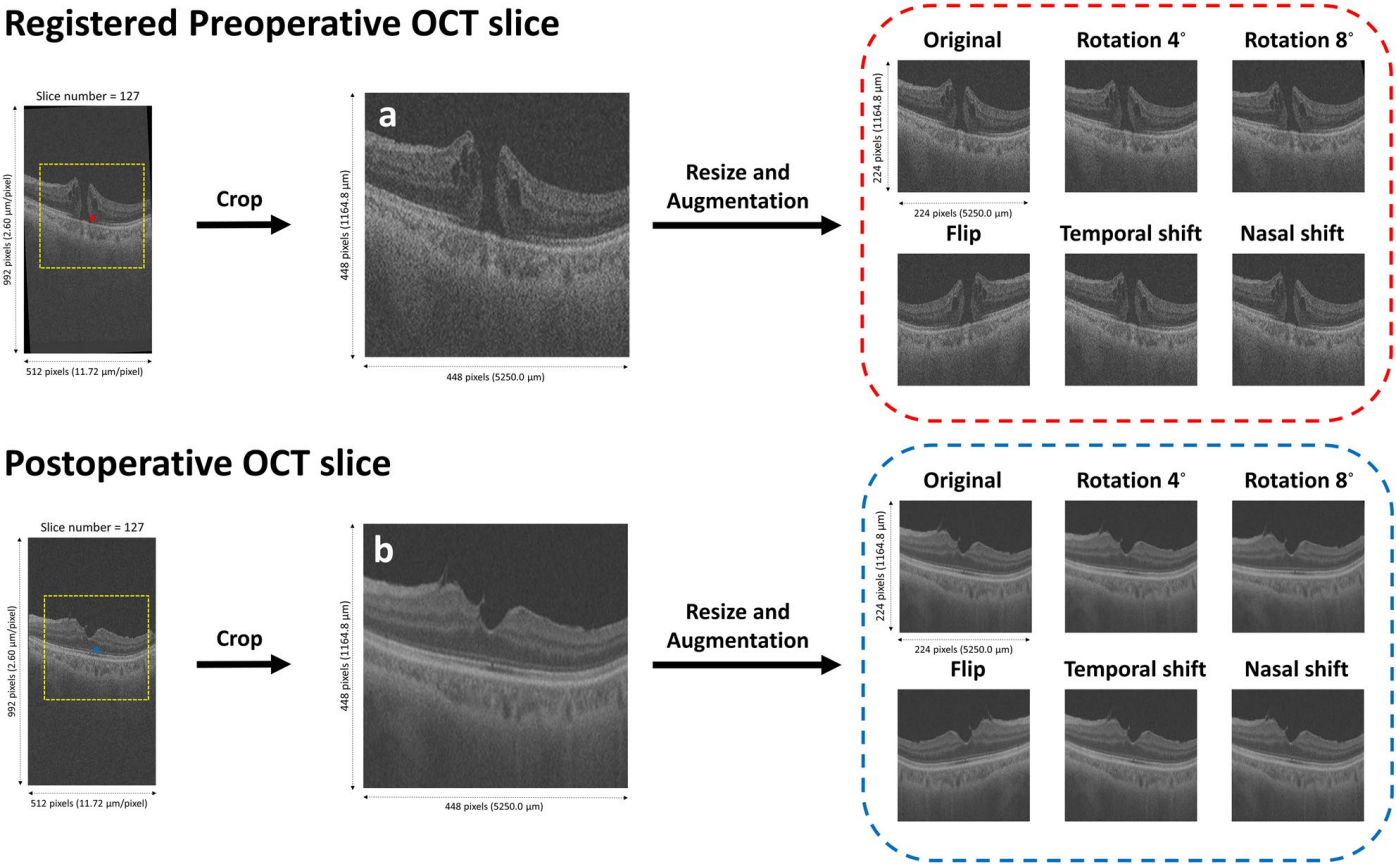
Preprocessing and augmentation of the training dataset. The preprocessing of the optical coherence tomography (OCT) images corresponding to the 127^th^ slice for the 79^th^ patient is included in the training dataset. The center of mass (COM) coordinate is extracted from preoperative OCT image. (**a**) A square area with a resolution of 448 pixels centered on the COM is cropped and resized to 224 square pixels using the LANCZOS4 interpolation method and designated as the original image. Five images are created by augmenting the original image, flipping horizontally, rotating counterclockwise by 4°and 8°around the COM, and shifting it by 15 pixels to the temporal and nasal sides. These images are then saved as augmentation data (red dotted line) and input into the generative deep learning model (GDLM). For the postoperative data, the same coordinate (blue asterisk) from the preoperative COM is used for the subsequent process, which is the same as for the preparation of the preoperative data. Consequently, (**b**) the original image and five augmented postoperative images (blue dotted lines) are designated as the ground truth for the GDLM. In order to allocate cases to each set randomly, we assigned consecutive integers to all cases in chronological order, following the sequence of prior surgical cases. Subsequently, we generated non-repeating random integers within Python software. The cases corresponding to randomly generated numbers were allocated in a 4:1:1 ratio, first to the training set and then to the validation and test sets. Multiple image pairs could come from the same volumetric scan, leading to a high correlation between adjacent image pairs and condition vectors. To minimize the correlation in the training dataset and address the imbalance in conditions, we utilized the WeightedRandomSampler modules in the process of loading training data sets (https://pytorch.org/docs/stable/_modules/torch/utils/data/sampler.html#WeightedRandomSampler. Accessed 21 January 2023).

For supervised learning the pairs of preoperative and postoperative slices were prepared according to the following sequence: An image with a resolution of 448 × 448 pixels was cropped, centered on the center of mass, and then resized by 50%. Only the 200 slices centered on the fovea were selected. Consequently, paired OCT images of 224 × 224 pixels (vertical 1164.8 μm and horizontal 5250.0 μm) were extracted from the volumetric OCT dataset for each patient. Augmentation techniques were then applied to the training set. This process increased the overall training data by six-fold (Fig. 2). All postoperative OCT images were designated as ground-truth OCT (GT-OCT) images. Since the foveal morphology and macular deformation vary according to the distance from the foveola^22^, different conditions were adopted for each image slice (See Supplementary Figure S2).

### Structure of generative deep learning model

**Figure 3:**
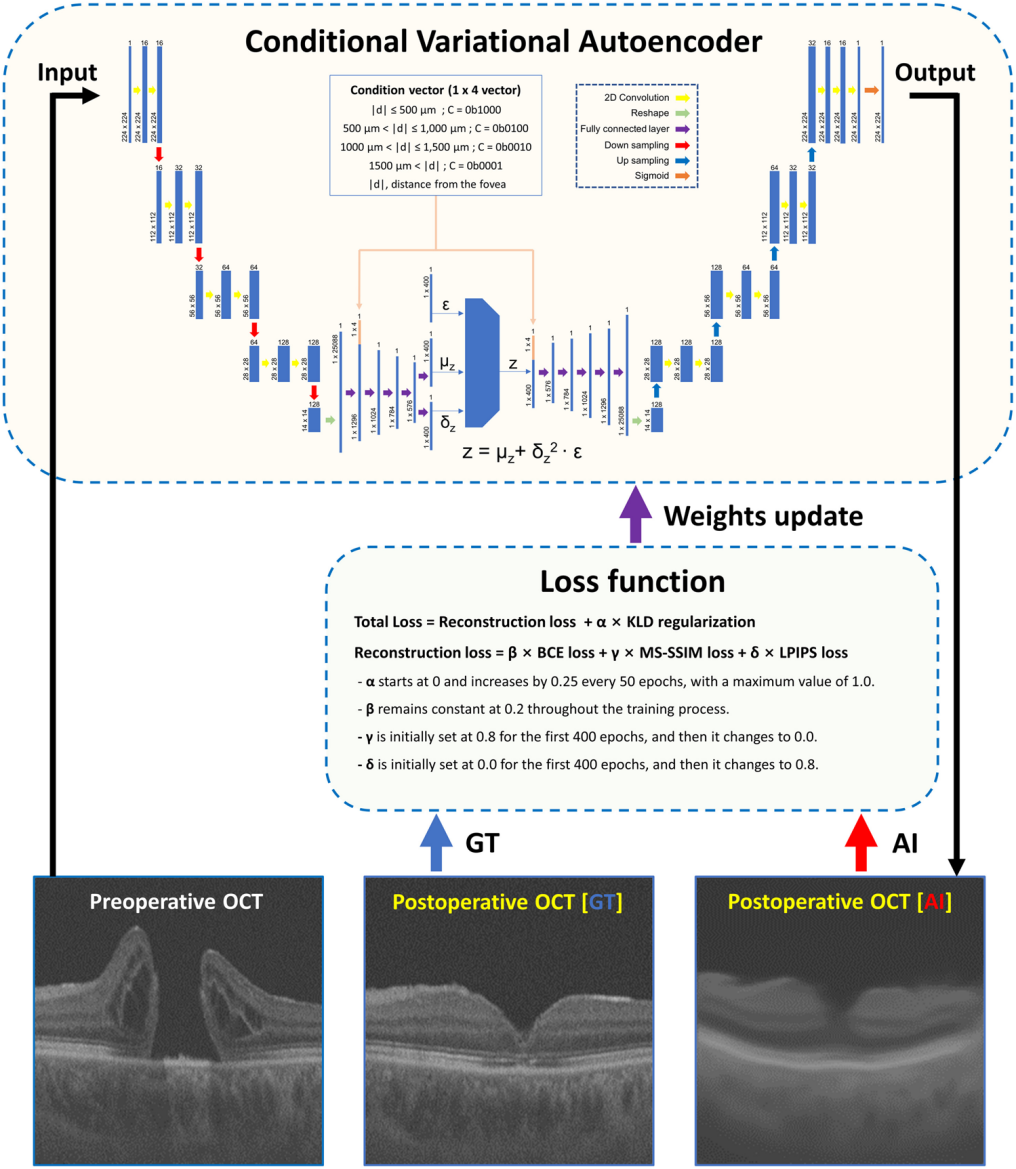
Architectural overview and training scheme and environments of a conditional variational autoencoder for optical coherence tomography image generation. The conditional variational autoencoder (VAE) architecture comprises an encoder, sampler, decoder, and a loss function unit. The encoder includes a four-layer two-dimensional (2D) convolutional neural network (CNN) and a five-layer one-dimensional (1D) fully connected network (FCN). The 2D CNNs in the encoder utilize a kernel size of 3, stride of 1, and padding of 1, with the number of channels doubling in subsequent layers. Each layer is followed by batch normalization, leaky rectified linear unit (LReLU) activation, dropout, and maximum pooling to prevent overfitting. The output of the 2D CNNs is transformed into a 1D vector and fed into the FCNs. The FCNs output two 1D vectors representing the mean (μ) and log-variance (log-var) vectors, with the first layer incorporating a condition vector. The sampler employs variational inference and a reparameterization trick to extract a 1D latent vector (z) from the encoder output. The decoder is designed in the reverse order, featuring five-layer FCNs, a four-layer 2D CNN decoder, and a sigmoid output layer. The CNNs in the decoder apply the nearest neighbor unpooling technique, which fills in the pixels surrounding the input data with the same value. During training, the VAE weights are updated to minimize the loss function comprising the reconstruction error and the Kullback–Leibler divergence (KLD) regularization. The reconstruction error incorporates binary cross-entropy with multiscale structural similarity (MS-SSIM) loss up to 400 epochs and then switches to learned perceptual image patch similarity (LPIPS) loss. A warm-up technique gradually increases the weight of the KLD regularization term to prevent latent vector deactivation. Network training is conducted on a Windows 11 operating system (version 22H2; Microsoft, Redmond, WA, USA) using the Jupiter Notebook platform. The CUDA version 11.8, PyTorch version 2.1.0, and OpenCV version 4.7.0 libraries are utilized. Additionally, network evaluation is performed in the same environment.

Conditional VAE was used for the GDLM and was designed to receive preoperative OCT image slices and condition vectors. This artificial intelligence (AI) model can generate postoperative OCT (AI-OCT) image slices and comprises four units: encoder, sampler, decoder, and loss function unit. In Fig. 3, these structures are explained in detail. The loss function unit calculated the difference between the AI-OCT images and the GT-OCT images and updated the weights of the GDLM to reduce this difference. In the late stage of the training process, perceptual loss functions were employed to generate fine AI-OCT images (See Supplementary Figure S3)^24,25^. The training process ended with 600 epochs and stopped if overfitting was detected. The epoch with the smallest loss was determined using the validation sets, and the weights of this epoch were loaded into the GDLM and used to evaluate the test set.

### Verification for accuracy and validity of generative deep learning model

For quantitative assessment, a representative pair of preoperative and postoperative cross-sectional OCT slices was selected from each test volumetric OCT dataset using the following steps: (1) The RPE surface from both preoperative and postoperative volumetric OCT data were extracted in each case within the test set. (2) Similar to the training set, we aligned the preoperative 3D volumetric OCT data with the RPE surface of the postoperative volumetric OCT through rotation and translation for registration. (3) The 128th OCT slice from the registered preoperative volumetric OCT was fed into the GDLM. (4) The 128th slice from the postoperative volumetric OCT was chosen as the GT-OCT slice. (5) The GDLM's output was designated the postoperative predicted OCT (i.e., AI-OCT) slice.

The quality of the AI-OCT image slices was assessed using three metrics: (1) image quality score, (2) agreement of the generated neurosensory retinal area, and 3) the learned perceptual image patch similarity (LPIPS) score. The image quality score ranges from 0 to 10, with one point assigned for each of the 10 distinct layers—the nerve fiber, ganglion cell, inner plexiform, inner nuclear, outer plexiform, outer nuclear layer, ELM, EZ, RPE, and choroidal vasculature in the AI- and GT-OCT image slice. A score of 0 was assigned when each retinal layer was not distinguishable or absent. To independently measure the image quality score, H.J.K. separated the AI-OCT images and GT-OCT images and distributed them to S.H.P. in PNUYH and J.H. in PNUH, respectively. If the judgment of the image quality score for each slice was ambiguous, the corresponding author provided the final decision.

**Figure 4:**
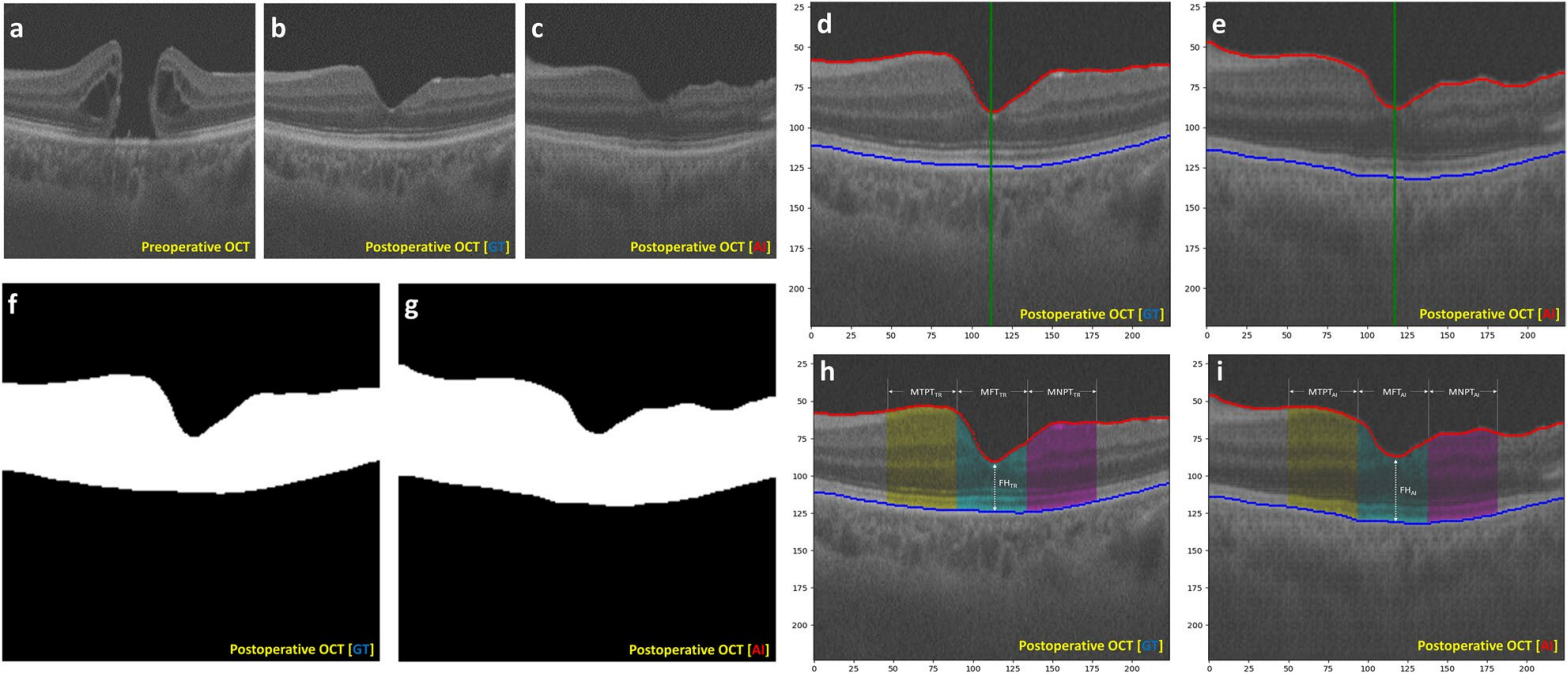
Quantitative assessment of predictive artificial intelligence optical coherence tomography image for full-thickness macular hole surgery. (**a**) A cross-sectional optical coherence tomography (OCT) slice of a full-thickness macular hole (FTMH) before surgery. (**b**) The postoperative 6-month OCT slice paired with slice A is regarded as the ground truth (GT) image. (**c**) The GDLM generates a predictive artificial intelligence (AI) OCT image. (**d**, **e**) The internal limiting membrane (red line) and retinal pigment epithelium layer (blue line) are accurately identified and superimposed onto the OCT slices. (**f**,** g**) White areas within the neurosensory retinal region are assigned TRUE, whereas other black areas are labeled FALSE to compare each pixel in the AI- and GT-OCT images. In this case, accuracy and F1 score are 95.2% and 0.901, respectively. (**h**, **i**) Foveolar height is measured at the foveola. Mean foveal thickness is defined as the mean retinal thickness corresponding to 500 μm on each side of the foveola (cyan region). Mean nasal and temporal parafoveal thickness is defined as the mean value of the retinal thickness corresponding to the section from 500 to 1500 μm in the nasal (yellow region) and temporal (magenta region) directions centered on the foveola.

The accuracy, precision, recall, and F1 scores were calculated to determine the concordant area of the neurosensory retina by binarizing and comparing the AI- and GT-OCT image slices (Fig. 4A–G). Manual corrections were applied to address boundary errors, and agreement metrics were extracted from all test sets. LPIPS score is one image similarity indicator that employs pre-trained image classification networks to evaluate the similarity between two images^25^. The comparison between AI- and GT-OCT image slices included assessments of LPIPS scores. A value close to 0 indicates a higher perceptual similarity between the two images^25^.

We performed Bland–Altman analyses to assess the agreement of foveolar height (FH), mean foveal thickness (MFT), and mean nasal/temporal parafoveal thickness (MNPT/MTPT) measured in AI- and GT-OCT (Fig. 4H,I). ELM restoration and EZ recovery were analyzed by assessing the accuracy and recall metrics between the two slices. Considering that the surgical procedures of the inverted ILM flap and conventional ILM peeling have different impacts on surgical outcomes and macular morphology^4^, the performance of the GDLM was quantitatively tested for two surgical techniques in the test set.

### Comparison of synthetic capabilities of various generative deep learning models

To our knowledge, there is yet to be any research or datasets utilizing GDLMs for synthesizing postoperative OCT images about FTMH. A few studies conduct OCT image synthesis to predict postoperative or post-therapeutic macular anatomy for other retinal diseases, such as wet age-related macular degeneration, retinal vein occlusion, and epiretinal membrane^26–28^. These studies employed GAN-based GDLMs, specifically the Pix2Pix and Pix2PixHD models. CycleGAN has been applied to address the variability of OCT images across different devices^29^. Among various VAE models, the nouveau VAE (NVAE) has showcased state-of-the-art performance on the MNIST dataset, evaluated by the bits per dimension metric^30^.

We compared the synthetic capabilities of our proposed model against various GDLMs. All GDLMs were trained up to 600 epochs using the same training dataset. We selected each model with the smallest loss, and synthetic capabilities were compared across 25 FTMH validation sets. The performance of each GDLM was evaluated with the LPIPS score, Fréchet Inception Distance (FID)^31^, and image quality score between postoperative AI-OCT and GT-OCT images.

### Statistical analyses

To analyze the data, the BCVA was converted to logMAR scale. Pearson's chi-square or Fisher's exact test is adopted to determine dependencies between two categorical variables. Continuous variables were compared using the Kruskal–Wallis test to compare each parameter among the three sets. Differences in FH, MFT, MNPT, and MTPT between AI- and GT-OCT slices were investigated using the Wilcoxon signed-rank test. Comparisons between ILM peeling and inverted ILM flaps were performed using the Mann–Whitney U test. A *P* value of < 0.05 was considered statistically significant. In Bland–Altman analyses to assess the agreement of macular morphology, upper and lower bounds of 95% limits of agreement (LoA) as a percentage difference using the Python pyCompare library (ver. 1.5.4). Bland–Altman plots were generated for each parameter, and we established an acceptable threshold if the 95% limits of LoA were both within ± 30%^32^. Univariate and multivariate logistic regression analyses were conducted to identify the baseline and intraoperative parameters that affect ELM and EZ disruption. Furthermore, we developed logistic regression models with training sets comprising statistically significant parameters and assessed its accuracy using test sets using the Python scikit-learn library (ver. 1.0.2).

### Ethics approval

Written informed consent was obtained from all participants. The study protocol and informed consent were approved by the Institutional Review Board of the PNUH and the PNUYH, and all research was conducted in accordance with the Declaration of Helsinki.

## Results

### Patients’ demographics

In total, 150 eyes with successfully closed FTMH met the inclusion criteria. Five surgeons performed the surgery on patients with an average age of 65.2 ± 9.0 years. The preoperative BCVA and CSMT were 0.773 ± 0.366 (20/119) and 320.5 ± 84.0 μm, respectively. Out of the total number of eyes, 47 (31.3%) were categorized as stage 2, 49 (32.7%) as stage 3, and 54 (36.0%) as stage 4 FTMHs, with a mean hole size of 448.0 ± 215.1 μm. Combined cataract surgery was conducted in 114 (76.0%) eyes. The inverted ILM flap technique was performed in 78 (52.0%) eyes, while ILM peeling in 72 eyes. The inverted ILM flap technique was performed in cases of larger hole size (538.8 ± 210.3 μm), compared to ILM peeling technique (349.7 ± 185.0 μm, *P* < 0.001). The postoperative BCVA and CSMT were significantly improved (0.393 ± 0.354 [20/49] and 259.6 ± 49.6 μm, respectively; *P* = 0.001 and *P* = 0.046, respectively). ELM restoration was achieved in 110 eyes (73.3%), and successful EZ recovery was observed in 101 eyes (67.3%). Eyes with complete restoration of ELM exhibited better BCVA than those without it (0.286 ± 0.267 vs. 0.688 ± 0.399, *P* < 0.001), and demonstrated greater BCVA improvement (0.457 ± 0.343 vs. 0.168 ± 0.222, *P* < 0.001). Additionally, eyes with EZ recovery exhibited better BCVA (0.289 ± 0.278 vs. 0.508 ± 0.394, *P* < 0.001) than those with failure of EZ recovery. All cases were randomly allocated to 100 training, 25 validations, and 25 test sets; none of the factors differed significantly across the three sets (Table 1).

**Table 1:** Characteristics of successfully closed full-thickness macular hole cases after surgery and comparison among training, validation, and test sets.

Factors	Total group (N = 150)	Training set (N = 100)	Validation set (N = 25)	Test set (N = 25)	*P* value
Baseline parameters					
Age (year)	65.2 ± 9.0	64.5 ± 9.5	67.2 ± 8.6	65.6 ± 7.5	0.253^a^
Male/female (N)	54/96	34/66	12/13	8/17	0.385^b^
Right/Left (N)	73/77	49/51	14/11	10/15	0.525^b^
Mean preoperative BCVA (logMAR)	0.773 ± 0.366	0.789 ± 0.374	0.744 ± 0.356	0.736 ± 0.353	0.746^a^
Mean preoperative CSMT (μm)	320.5 ± 84.0	322.5 ± 80.5	334.8 ± 81.7	298.3 ± 99.8	0.374^a^
Axial length (mm)	23.8 ± 1.5	23.8 ± 1.6	24.1 ± 1.3	23.7 ± 1.1	0.137^a^
Hole size (μm)	448.0 ± 215.1	445.0 ± 197.6	413.4 ± 230.9	494.8 ± 262.9	0.478^a^
FTMH Stage 2/Stage 3/Stage 4 (N)	47/49/54	30/36/34	11/7/7	6/6/13	0.269^b^
FTMH morphology (N) [M shape/U shape/W shape]	81/49/20	55/34/11	12/8/5	14/7/4	0.772^b^
Intraoperative parameters					
Combined surgery (N)	114	79	18	19	0.103^b^
ILM manipulation technique (N) [Peeling only/Inverted flap]	72/78	45/55	13/12	14/11	0.560^b^
Size of ILM peeling (Disc diameter)	3.2 ± 0.7	3.2 ± 0.7	3.1 ± 0.8	3.2 ± 0.7	0.779^a^
Surgeon (N) [Surgeon 1/2/3/4/5]	6/40/35/41/28	5/26/22/25/22	0/8/5/8/4	1/6/8/8/2	0.753^c^
ILM staining dye (N) [BBG/ICG]	76/74	56/44	10/15	10/15	0.182^b^
Tamponade materials (N) [Air/SF_6_]	77/73	50/50	15/10	12/13	0.628^b^
Postoperative parameters					
Postoperative BCVA (logMAR)	0.393 ± 0.354	0.397 ± 0.376	0.348 ± 0.229	0.420 ± 0.376	0.891^a^
Postoperative CSMT (μm)	259.6 ± 49.6	259.0 ± 43.8	270.2 ± 52.5	251.3 ± 66.3	0.674^a^
ELM restoration/disruption (N)	110/40	74/26	18/7	18/7	0.966^b^
Success/Failure of EZ recovery (N)	101/49	70/30	16/9	15/10	0.589^b^

BBG, brilliant blue G; BCVA, best-corrected visual acuity; CSMT, central subfield mean thickness; ELM, external limiting membrane; EZ, ellipsoid zone; FTMH, full-thickness macular hole; ICG, indocyanine green; ILM, internal limiting membrane; logMAR, the logarithm of the minimum angle of resolution; OCT, optical coherence tomography.

^a^Results of the Kruskall–Wallis test.

^b^Results of the Pearson’s chi-square test.

^c^Results of the Fisher’s exact test.

### Training GDLM and artificial intelligence OCT images

Of the 120,000 OCT image slice pairs in the training set, 25,800 pairs were set to condition vector 0; 25,200 pairs to condition vector 1; 25,800 pairs to condition vector 2; and 43,200 pairs to condition vector 3. As the number of epochs increased, the conditional VAE gradually reduced the loss, and the AI-OCT image slices resembled the GT-OCT image slices (See Supplementary Figure S3, S4, and Movie S5).

### Comparison of various GDLMs with validation set

Proposed conditional VAE adopting LPIPS loss showed the lowest LPIPS and the highest image quality scores compared with GAN-based GDLMs (See Supplementary Table S6). Supplementary Figure S7 depicted representative cases of GT- and AI-OCT images predicted by various GDLMs. The Pix2Pix model achieved the lowest FID score, but spatial deformity was observed in specific cases. CycleGAN was excluded because synthesized OCT images are almost identical to preoperative OCT images.

### Comparison of accuracy and validity between AI- and GT-OCT image slices

AI-OCT image slices from test sets had a mean image quality score of 9.80 ± 0.50, clearly distinguishing between the retinal layers and surrounding structures, and were no different from the image quality scores of GT-OCT image slices (9.96 ± 0.20). The accuracy of the agreement between AI- and GT-OCT image slices for retinal regions was 94.7 ± 2.0%, with a precision of 89.0 ± 6.6%, recall of 89.5 ± 4.9%, and F1 score of 0.891 ± 0.042. The mean LPIPS score was 0.135 ± 0.033. Eyes with ILM peeling (n = 14, 56.0%) exhibited higher accuracy (95.5% vs. 93.6%, *P* = 0.025), precision (91.4% vs. 85.9%, *P* = 0.038), and F1 scores (0.909 vs. 0.868, *P* = 0.018) compared to those with inverted ILM flaps (n = 11, 44.0%) (Table 2).

**Table 2:** Qualities and anatomical similarity between predictive and actual postoperative optical coherence tomography images.

Parameters	AI-OCT image	GT-OCT image	*P* value
All test set (N = 25)			
Image quality (0–10)	9.80 ± 0.50	9.96 ± 0.20	0.102^a^
Agreement for retinal area			
Accuracy (%)	94.7 ± 2.0		
Precision (%)	89.0 ± 6.6		
Recall (%)	89.5 ± 4.9		
F1 score	0.891 ± 0.042		
LPIPS score (0–∞)	0.135 ± 0.033		
FH (μm)	228.2 ± 51.8	233.4 ± 70.0	0.596^a^
MFT (μm)	271.4 ± 35.5	273.3 ± 55.7	0.819^a^
MNPT (μm)	316.6 ± 35.2	311.2 ± 34.3	0.367^a^
MTPT (μm)	314.1 ± 32.7	309.7 ± 36.0	0.989^a^
ILM peeling group (N = 14)			
Image quality (0–10)	10.00 ± 0.00	10.00 ± 0.00	1.000^a^
Agreement for retinal area			
Accuracy (%)	95.5 ± 1.7		0.025^b^
Precision (%)	91.4 ± 4.5		0.038^b^
Recall (%)	90.6 ± 5.2		0.244^b^
F1 score	0.909 ± 0.034		0.018^b^
LPIPS score (0–∞)	0.126 ± 0.035		0.166^b^
FH (μm)	229.2 ± 53.6	236.6 ± 62.8	0.440^a^
MFT (μm)	273.5 ± 39.1	278.2 ± 45.5	0.470^a^
MNPT (μm)	319.0 ± 36.4	316.7 ± 39.2	0.683^a^
MTPT (μm)	315.2 ± 32.1	314.7 ± 26.2	0.683^a^
ILM Inverted flap group (N = 11)			
Image quality (0–10)	9.55 ± 0.69	9.91 ± 0.30	0.102^a^
Agreement for retinal area			
Accuracy (%)	93.6 ± 2.1		0.025^b^
Precision (%)	85.9 ± 7.7		0.038^b^
Recall (%)	88.2 ± 4.3		0.244^b^
F1 score	0.868 ± 0.041		0.018^b^
LPIPS score (0–∞)	0.146 ± 0.027		0.166^b^
FH (μm)	226.9 ± 51.9	229.3 ± 81.1	0.858^a^
MFT (μm)	268.7 ± 32.0	267.1 ± 68.4	1.000^a^
MNPT (μm)	313.7 ± 35.3	304.2 ± 27.1	0.374^a^
MTPT (μm)	312.6 ± 35.0	303.4 ± 46.2	0.657^a^

AI, artificial intelligence; ELM, external limiting membrane; ILM, internal limiting membrane; FH, foveolar height; GT, ground truth; LPIPS, learned perceptual image patch similarity; MFT, mean foveal thickness; MNPT, mean nasal parafoveal thickness; MTPT, mean temporal parafoveal thickness; OCT, optical coherence tomography.

^a^Results of the Wilcoxon signed-rank test between AI- and GT-OCT images.

^b^Results of the Mann–Whitney U test between the ILM peeling and inverted flap groups.

AI- and GT-OCT image slices revealed no statistically significant differences in the averages of FH (228.2 ± 51.8 vs. 233.4 ± 70.0 μm), MFT (271.4 ± 35.5 vs. 273.3 ± 55.7 μm), MNPT (316.6 ± 35.2 vs. 311.2 ± 34.3 μm), and MTPT (314.1 ± 32.7 vs. 309.7 ± 36.0 μm). No statistically significant difference was observed in retinal thickness between the ILM inverted flap and peeling groups (Table 2). The 95% LoA for all morphological parameters in both the test sets and the ILM peeling group remained within the range predefined by the cut-off value. However, in the case of the ILM inverted flap group, the upper 95% LoA for MFT exceeded 30% (See Supplementary Figure S8).

The logistic regression analysis revealed that a larger hole size was the sole factor that increased the risk of both ELM and EZ disruption among the baseline and intraoperative parameters. The ILM peeling group demonstrated a higher probability of EZ recovery (See Supplementary Table S9). These logistic regression models predicted ELM and EZ recovery accuracy rates at 84.0% and 76.0%, respectively.

**Figure 5:**
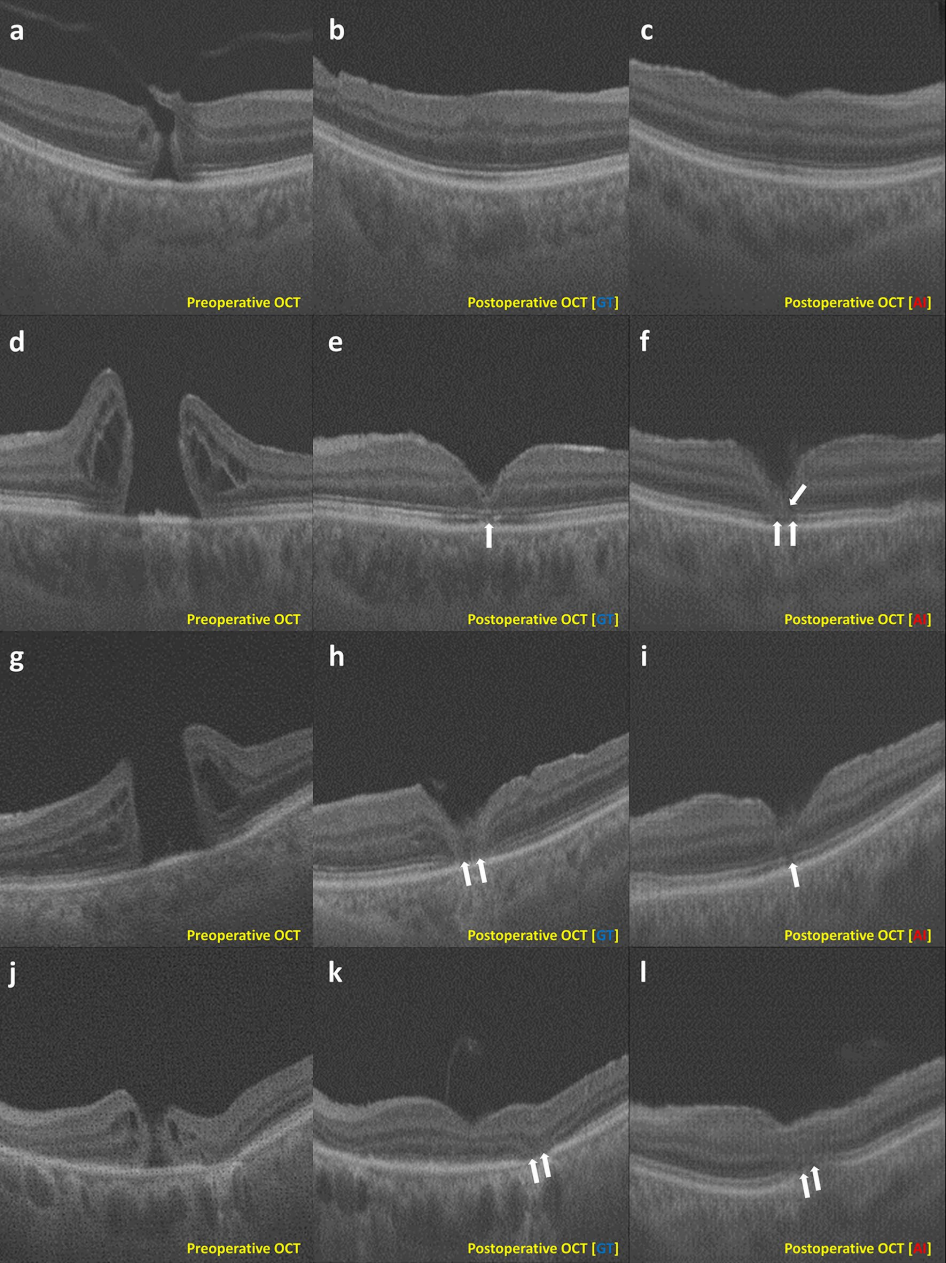
Representative cases with predicted and ground truth postoperative optical coherence tomography images using a generative deep learning model. The leftmost column depicts preoperative optical coherence tomography (OCT) images from the test set used as input for the generative deep learning model (GDLM). All conditions in this figure are zero since the OCT cross-section passes near the fovea. The middle column shows the postoperative ground truth (GT) OCT images (GT-OCT), and the rightmost column displays the postoperative OCT images predicted using the GDLM. (**a–c**) The images of the GT-OCT and predicted artificial intelligence (AI) OCT (AI-OCT) are highly similar. The F1 score for the binarized neurosensory retinal region is 0.949. Image **c**, generated by the GDLM, encompasses all retinal layers, resulting in a quality score 10. (**d–f**) Unlike in the GT-OCT image (**e**), the external limiting membrane (ELM) is disrupted due to glial proliferation in the AI-OCT image (**f**, white arrows). (**g–i**) In the GT-OCT image, the ellipsoid zone (EZ) is disrupted (**h**); also, EZ disruption is apparent in the AI-OCT image (**i**). However, ELM disruption in GT-OCT is observed as a continuous line in AI-OCT, categorized as ELM restoration. (**j–l**) The ELM and EZ are disrupted in both images (**k** and **l**, white arrows).

The accuracy of ELM restoration using GDLM was 88.0%, with recall rates of 94.4% for successful restoration and 71.4% for cases of restoration failure. For the EZ recovery, the GDLM achieved an accuracy of 92.0%, with recall rates of 100.0% for successful recovery and 80.0% for failures. The accuracy of GDLM model was superior to statistical analysis in prediction for postoperative foveal microstructure. The confusion matrices for predicting the ELM and EZ restoration are summarized in Supplementary Figure S10. Fig. 5 and Supplementary Figure S11 show representative cases of AI- and GT-OCT images.

## Discussion

This study introduces the GDLM for predicting postoperative macular structures based on preoperative OCT images using a modified conditional VAE architecture. The AI-OCT images effectively depicted the distinct retinal layers as well as RPE and choroid. The high accuracy and F1 score indicated strong agreement with the neurosensory retinal region between the AI- and GT-OCT images. The retinal thickness did not differ between AI- and GT-OCT images. The accuracy of restoring the ELM and EZ was 85% or higher. These findings indicated that the proposed GDLM could generate high-quality AI-OCT images, similar to the actual postoperative OCT images. The average FH and MFT of AI-OCT images were comparable to those of GT-OCT images with a difference of not more than 5.2 μm, which is within a one-pixel level of mean differences. Bland–Altman analyses of the four thickness profiles showed that the upper and lower bounds of the 95% LoA did not exceed 30%. This indicates that the predicted thickness profiles can be considered acceptable as a new technique^32^.

Wakabayashi et al. identified that the ELM status at 3 months was related to the BCVA 1 year after surgery^12^. Further, ELM recovery may be a prerequisite for the EZ recovery^13^. However, presenting the postoperative ELM status as a simple probability value may lack persuasiveness for patients and physicians. Postoperative AI-OCT images generated by the GDLM could provide an intuitive prediction of ELM restoration by determining the presence of a continuous bright line corresponding to the ELM in a 2D OCT image. The GDLM exhibited a significant resemblance to the GT-OCT images in eyes with ILM peeling, accurately reproducing retinal structures compared to those with the inverted ILM flap. Following MH surgery, the inverted flap group experiences more glial proliferation around the fovea than the ILM peeling group^33^. Consequently, such foveal proliferation results in diversity for the ILM inverted flap group^12,34^. This diversity can potentially hinder the accurate prediction of macular structures by the GDLM in the ILM inverted flap group. Nevertheless, the proposed model effectively predicted not only other retinal thickness profiles but also ELM and EZ restoration in eyes experienced with ILM inverted flaps. The recall rates for ELM and EZ restoration failure were at 71.4% and 85.6%, respectively. This observation can be elucidated as follows: ELM and EZ disruption present as diverse signal intensities in postoperative OCT images, potentially introducing inaccuracies into the generative model. For instance, PRL defects may exhibit low signal intensity alongside outer foveal defects or moderate hyperreflective lesions resulting from glial cell proliferation, even in the ILM peeling group^35^. In addition, the diminished recall rate might be attributed to the limited number of ELM and EZ disruption cases. Additional training sets of ELM and EZ disruption cases may enhance the recall rate.

We employed a conditional VAE that was trained on foveal OCT images as well as parafoveal OCT images corresponding to different conditions. The OCT training set for conditions other than condition 0, located away from the fovea, was more than three times larger than that for condition 0. The parafoveal OCT images revealed detailed retinal layers and surrounding structures. This approach enabled the GDLM to effectively represent specific retinal layers and predict various anatomical conditions for both the foveal and parafoveal regions from limited training sets. The realistic generation of parafoveal OCT images using the proposed GDLM is depicted in Supplementary Figure S11.

Studies have been conducted on the use of discriminative DL models in predicting VA or surgical outcomes in FTMH using preoperative cross-sectional OCT images. Obata et al. found the DL model to be more accurate in predicting VA than multivariate linear regression with baseline factors^16^. In contrast, Lachance et al. observed no significant improvement in visual prediction by adding clinical findings to a CNN-based DL model^36^. The use of preoperative cross-sectional OCT images in DL models influencing postoperative VA can lead to varied results. Predicting postoperative macular structures could be key to forecasting postoperative VA^11,12^. Xiao et al. introduced a DL model for predicting postoperative hole closure at one month using preoperative OCT images and clinical data^15^. This model achieved an accuracy of over 80% in predicting MH closure. However, their successful closure rate was less than 70%, significantly lower than the primary hole closure rate of over 95% reported in recent studies^7,8^. The prerequisite for using our GDLM model is knowledge of MH closure, which is impossible to determine before surgery. Nonetheless, since cases where idiopathic FTMH fails to close after the primary surgery are rare, these limitations will only affect a small number of patients.

In GAN-based GDLMs, spatial deformity (Supplementary Figure S7J and S7K) occurs in small training sets or spatial misalignment of image pairs^18^. Achieving exact structural and spatial alignment in image-to-image transformation with conditional GANs is crucial, often requiring further registration during preprocessing to ensure high-quality images^37,38^. However, the newly remodeled and migrated neural structure around the fovea makes accurate structural alignment impossible following FTMH surgery. Therefore, we designed a new VAE-based GDLM to predict postoperative OCT images in FTMH patients, as GAN-based GDLMs encounter these drawbacks. Considering the drawbacks of GANs, such as instability in training, mode collapse, and spatial deformity when generating images^18^, the stability of VAEs is more suitable for medical applications.

This study had other several limitations, including its small sample size and retrospective nature. The GDLM exclusively employed preoperative OCT data during the training phase, and did not utilize clinical factors which can affect the anatomical status of the macula after FTMH surgery. Chronicity, stage of MH, and preoperative VA are major factors affecting postoperative anatomical and functional status^39,40^. Incorporating these factors as supplementary condition vectors or integrating them with DL models holds the potential to enhance their performance. The proposed GDLM could not predict postoperative visual prognosis. Future research is required to develop an advanced DL model that can infer visual prognosis from predicted OCT images.

Despite these limitations, the proposed GDLM can serve as an explainable AI technique by providing predicted OCT images and anatomical profiles surrounding the macula rather than relying on simplistic measurements, such as VA or hole closure, as utilized in conventional CNN-based DL model. Further, the introduced GDLM can be applied to various fields where the anatomical status of the macula after intervention needs to be predicted through OCT images, not limited to FTMH patients but also in all retinal conditions requiring the involvement of retinal specialists. Therefore, this GDLM will serve as a significant initial step in post-intervention OCT prediction. This study is anticipated to provide valuable clinical assistance to patients and ophthalmologists in assessing postoperative prognosis. Cases that predict ELM or EZ defects in AI-OCT images may result in a poor visual prognosis. In such cases, strategies to enhance their integrity can be considered before or during surgery, such as advising early surgery to the patient or considering ILM inverted flap techniques instead of ILM peeling^6,41,42^.

In conclusion, the proposed GDLM demonstrated the ability to generate realistic and accurate predictions of postoperative OCT images. It successfully captured detailed retinal structures with a high degree of regional agreement and has the potential to provide valuable clinical insights by forecasting the restoration of ELM and EZ conditions closely related to postoperative prognosis. Sharing these predictive OCT images with patients scheduled for vitrectomy allows us to directly inform them about the surgical benefits and goals.

### Supplementary Information

Supplementary Information.

Supplementary Movie S5.

## Data Availability

The datasets for this study are protected patient information. Some data may be available for research purposes from the corresponding author upon reasonable request.
