# Supplementary material for: Accuracy of generative deep learning model for macular anatomy prediction from optical coherence tomography images in macular hole surgery
Source: Sci Rep. 2024 Mar 22;14:6913. doi: 10.1038/s41598-024-57562-5 (PMC10959933; doi:10.1038/s41598-024-57562-5)
Supplement: Supplementary file 1 — Supplementary Information. [file 41598_2024_57562_MOESM1_ESM.pdf]

Supplementary Table S1. Inclusion and exclusion criteria of the patients with idiopathic full-thickness macular hole

Criteria	Number of cases (N)
**Inclusion criteria**	
Consecutive FTMH patients who underwent PPV, ILM peeling, and FAX between Jan. 2018 and Dec. 2022 and taken by 6 × 6 mm^2 macular volume scans via SS-OCT	275
**Exclusion criteria**	
Failure cases of MH closure after the first vitrectomy	7
The latter case of bilateral FTMHs	17
Secondary FTMH	16
MH with retinal detachment	27
Category 2 or higher grade of pathologic myopia	11
ILM autologous insertion	8
Silicone oil tamponade	3
Previous glaucoma surgery	4
Previous vitrectomy history	8
Low signal intensity on volumetric OCT data (Image quality score < 60)	10
Discontinuation of RPE layer	14
**Total cases for training, validation, and test for GDLM**	150

FAX = fluid-air exchange, FTMH = full-thickness macular hole, ILM = internal limiting membrane, MH = macular hole, OCT = optical coherence tomography, PPV = pars plana vitrectomy, RPE = retina pigment epithelium, SS-OCT = swept-source optical coherence tomography.

Supplementary Figure S2. Labeling condition vector based on preoperative optical coherence tomography slices.

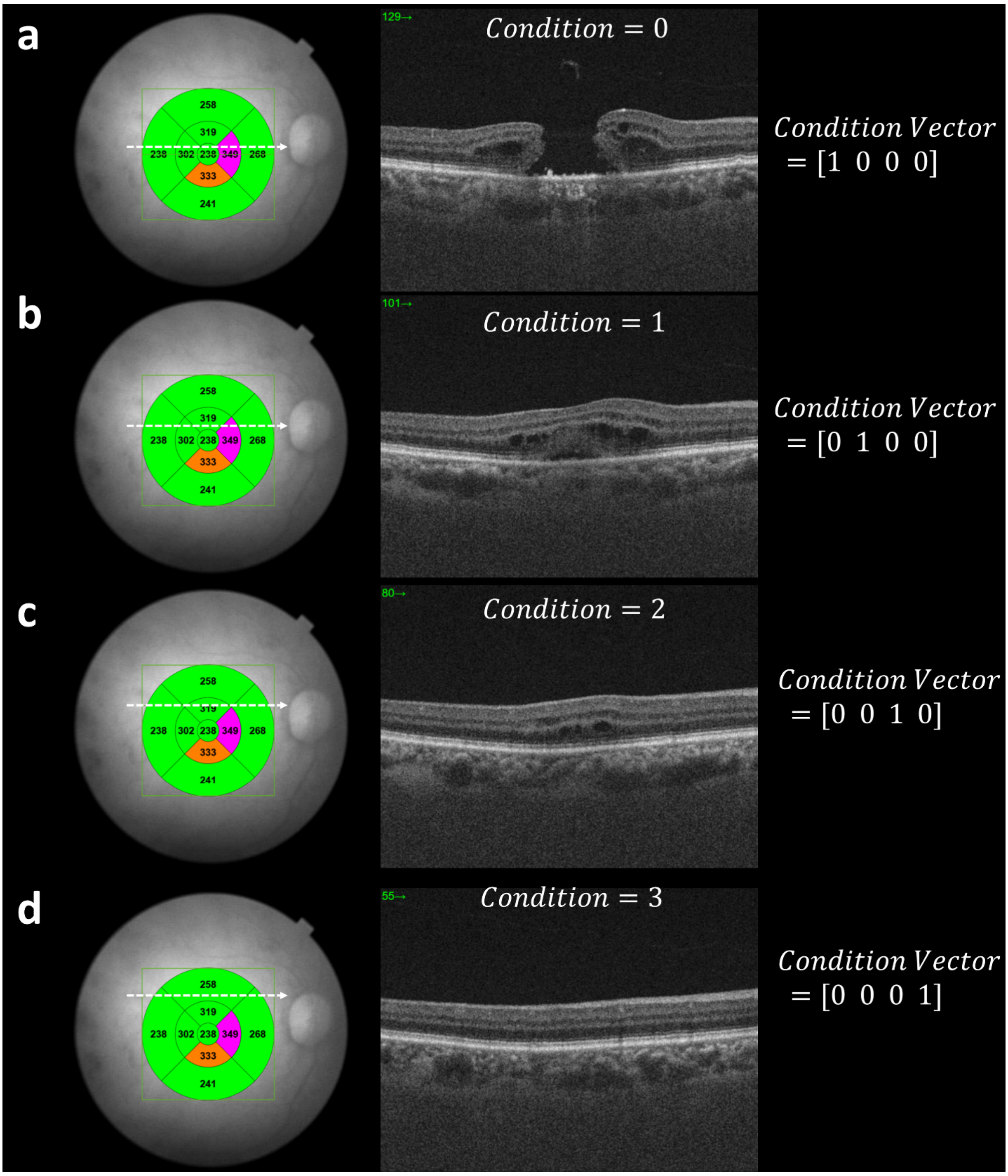

Macular volume scans obtained using optical coherence tomography (OCT) contain slices ranging from 1 to 256. Different sets of condition vectors are established at intervals of 500 μm from the fovea in the horizontal OCT image of each volumetric data point to indicate the degree of distance (white dotted arrows). The condition value was increased by 1 from 0 to every 500 μm away from the foveola. (**a**) The 129th slice, immediately adjacent to the slice passing through the fovea and slices within 500 μm from the fovea, is assigned a condition of 0. (**b**) The 101st slice, 633 μm (27 slices) away from the fovea, is assigned a condition of 1, corresponding to the slices between 500 and 1,000 μm from the fovea. (**c**) The 80th slice, positioned 1,125 μm away from the fovea, is assigned a condition of 2, corresponding to the slices between 1,000 and 1,500 μm from the fovea. (**d**) All slices located beyond 1,500 μm from the fovea are assigned a condition of 3. The 4-bit condition vector is labeled for each slice using the 1-of-K scheme (K = 3) applied to each condition.

Supplementary Figure S3. Changes in the loss of generative deep learning model throughout the training phase.

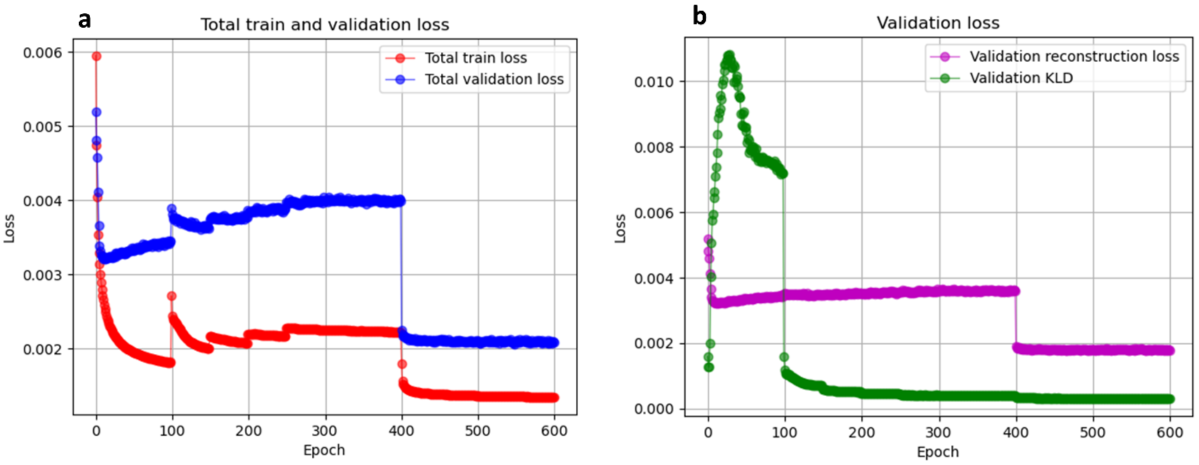

Training required 5,172 min for 600 epochs, and loss values were visualized in a graph throughout the training. Early discontinuation was not observed. (**a**) Graph depicting the changes in the loss of training (red) and validation (blue) sets over 600 epochs. This loss comprises the reconstruction loss and Kullback–Leibler divergence (KLD) regularization. Initially, only the reconstruction loss is used as the autoencoder mode for the first 100 epochs, after which a warm-up technique is applied that gradually increases the proportion of the KLD regularization in the loss function by 25% for every 50 epochs. The reconstruction loss is composed of a linear combination of binary cross-entropy (BCE) loss and multiscale structural similarity up to epoch 400 and is later replaced with a linear combination of BCE loss and learned perceptual image patch similarity. The minimum validation loss was attained at epoch 533 and was employed in the GDLM for the evaluation of the test set. (**b**) The plot shows the reconstruction (magenta) and KLD (green) losses for the validation set. After 100 epochs, the KLD loss steadily decreases. At epoch 533, the average reconstruction and KLD losses are 1.79×10^{-3}, and 0.31×10^{-3}, respectively.

Supplementary Figure S4. Optical coherence tomography images predicted by the generative deep learning model as training progresses.

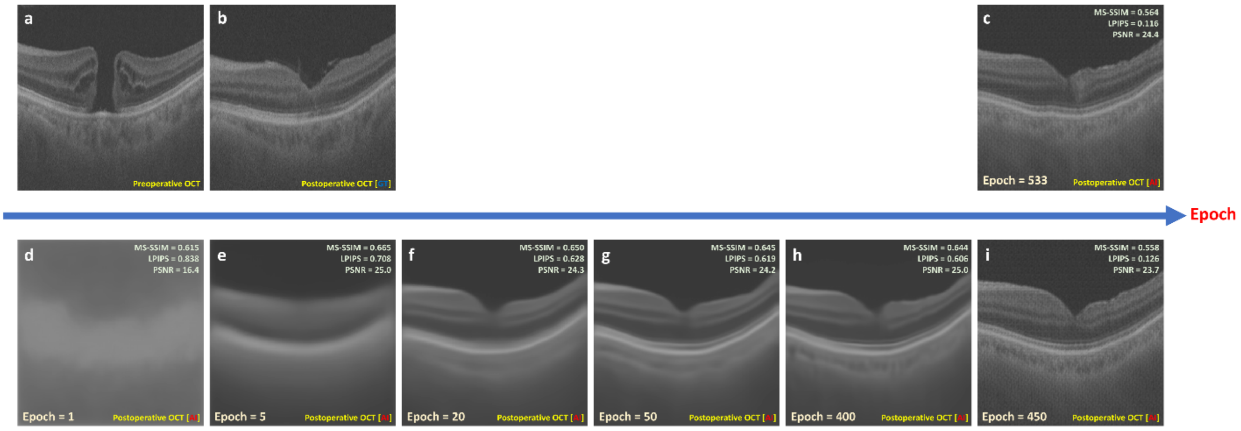

(**a**) Preoperative cross-sectional optical coherence tomography (OCT) slice. (**b**) Postoperative OCT image in the validation set as the ground truth (GT) OCT slice. (**c**) Predicted OCT slices by generative deep learning model (GDLM) trained for 533 epochs. (**d**) At epoch 1, GDLM shows faint bright boundaries in the retinal region. (**e**) By epoch 5, GDLM distinguishes the retinal pigment epithelium and the vitreous-retina border, with improved multiscale structural similarity (MS-SSIM) and peak signal-to-noise ratio (PSNR), and reduced learned perceptual image patch similarity (LPIPS), indicating closer resemblance to GT image. However, the GDLM cannot differentiate between the choroid and sclera. (**f**) At 20 epochs, the GDLM can distinguish choroidal vessels, and most retinal layers begin to separate. (**g**) At 50 epochs, the external limiting membrane and choroidoscleral junction are identified. (**h**) At 400 epochs, all retinal structures are apparent as distinct layers, albeit somewhat blurry. (**i**) Post-epoch 401, the LPIPS loss nears zero, yielding a more realistic image compared to the GT image. However, MS-SSIM and PSNR decrease, indicating that these two metrics do not reflect perceptual similarity as effectively as LPIPS.

Supplementary Movie S5. Changes in optical coherence tomography predictions with progressive training of generative deep learning models.

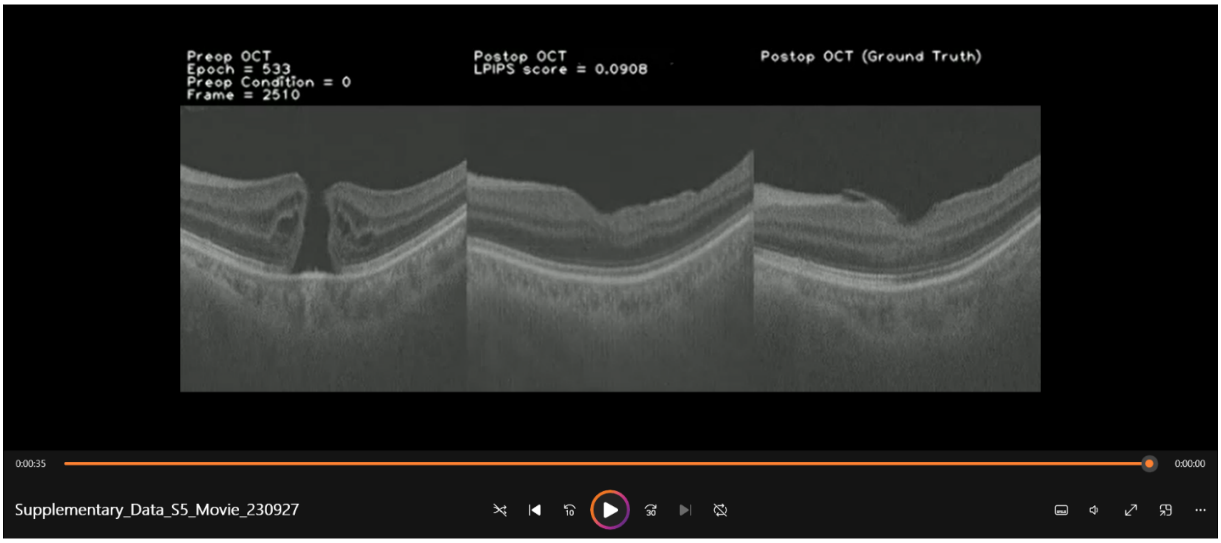

Changes in the postoperative optical coherence tomography slices, as predicted by the GDLM, were converted into video clips.

Supplementary Table S6. Performance of various generative deep learning models in postoperative optical coherence tomography image prediction of idiopathic full-thickness macular hole

Types of GDLMs	LPIPS score (↓)	FID (↓)	Image Quality Score (↑)
**GAN-based GDLMs**			
Pix2PixHD	0.157	123.121	9.24
Pix2Pix	0.162	**111.534**[a]	9.80
CycleGAN	0.200	187.151	10.00[b]
**VAE-based GDLMs**			
NVAE	0.571	236.114	9.48
CVAE with BCE + MS-SSIM loss	0.609	225.036	9.60
CVAE with BCE + MS-SSIM / LPIPS loss	**0.151**[a]	170.592	**9.88**[a]

BCE = binary cross-entropy, CVAE = conditional variational autoencoder, FID = Fréchet inception distance, GAN = generative adversarial network, GDLM = generative deep learning model, LPIPS = learned perceptual image patch similarity, MS-SSIM = multiscale structural similarity, NVAE = nouveau variational autoencoder, VAE = variational autoencoder.

[a] = The best scores are highlighted in bold.

[b] = Although CycleGAN showed the highest image quality score, the synthesized postoperative optical coherence tomography (OCT) images were identical to the preoperative OCT images. Therefore, CycleGAN was excluded from the comparison.

Supplementary Figure S7. Comparison of Postoperative Optical Coherence Tomography Images Using Various Generative Deep Learning Models.

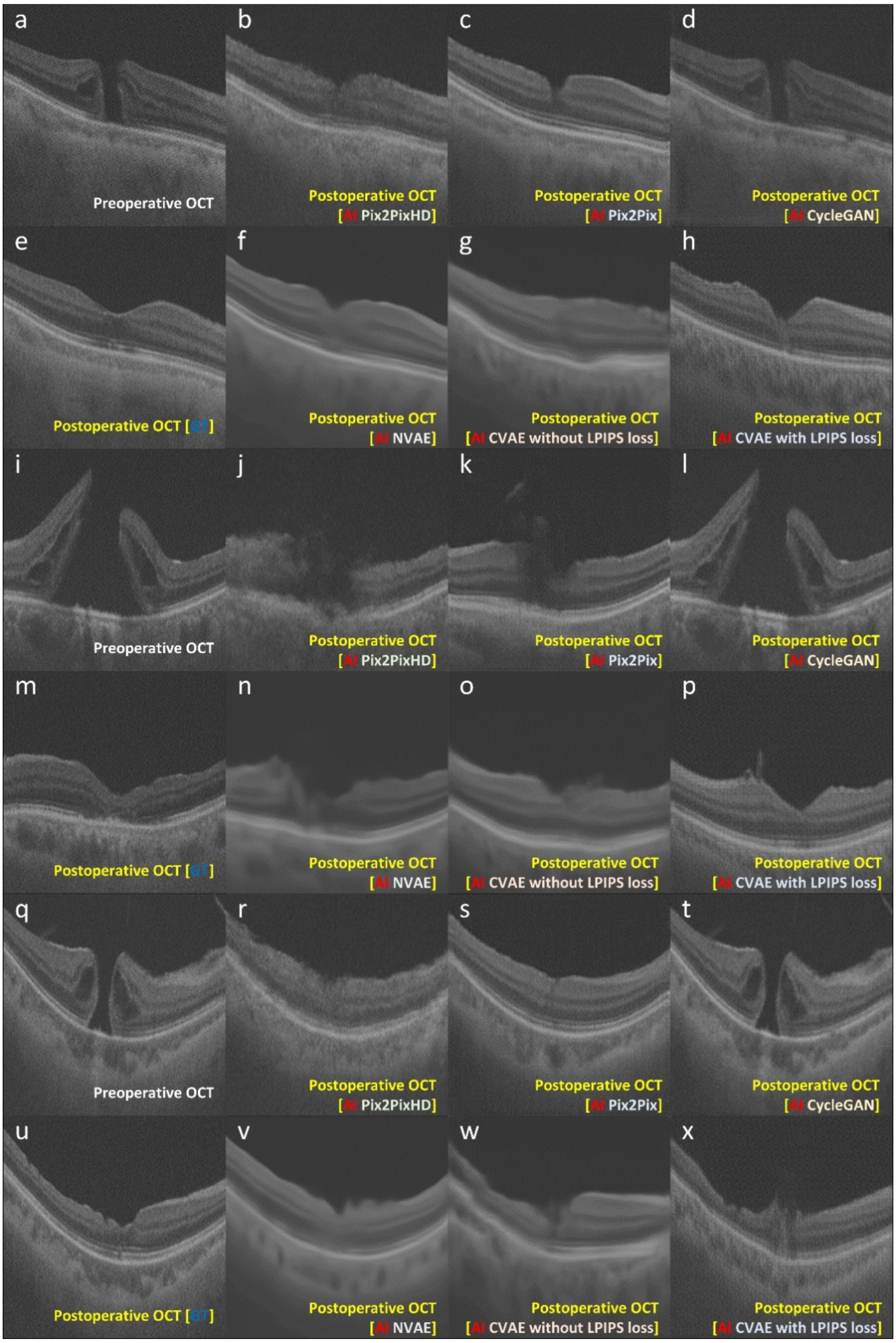

(**a-h**) Pre-/Postoperative OCT images from the validation set No. 8 synthesized by various GDLMs (**a**) The preoperative optical coherence tomography (OCT) image of the full-thickness macular hole (FTMH) is input into the various generative deep learning models (GDLMs). (**b-d**) Postoperative OCT images, predicted by each GAN-based GDLM, are noted with the model type at the bottom right. (**b, c**) The Pix2PixHD model synthesizes the image with higher spatial resolution in each retinal layer compared to the Pix2Pix model. (**d**) The OCT image predicted by CycleGAN is sharp but almost does not alter the spatial information of the preoperative OCT image (**a**), and it is nearly identical to the preoperative OCT image. (**e**) The real postoperative OCT image shows that the external limiting membrane (ELM) is continuous at the fovea but discontinuous in the ellipsoid zone (EZ). (**f-h**) Variational autoencoder (VAE)-based GDLMs produce synthetic postoperative OCT images. (**f**) The nouveau VAE (NVAE) image is blurrier than those generated by the GAN-based models but successfully predicts the EZ discontinuity. (**g**) Without adopting learned perceptual image patch similarity (LPIPS) loss to the proposed model, the output OCT image is similar to NVAE but still blurry. (**h**) After adopting LPIPS loss to our conditional VAE, the model achieves clarity as the GAN-based images also accurately depict EZ disruption.

(**i-p**) Pre-/Postoperative OCT images from the validation set No. 18. (**i**) A large diameter macular hole is present in the preoperative OCT. (**j, k**) Due to the spatial deformity of GAN, the Pix2PixHD and Pix2Pix models lose the fovea and adjacent retinal layers in synthesized postoperative OCT images. (**l**) The image produced by CycleGAN closely resembles the preoperative OCT image. (**m**) A foveal notch and EZ disruption are noted on the ground-truth (GT) postoperative OCT image. (**n-p**) In synthetic images based on VAE, spatial deformities were not observed. (**n**) The macular morphology of the OCT image generated by the NVAE resembles that of the GT OCT image (**m**)

but is still blurry. (**p**) Our proposed model predicted postoperative EZ attenuation but did not accurately depict ELM discontinuation.

(**q-x**) Pre-/Postoperative OCT images from the validation set No. 23. (**q**) Preoperative OCT images display myopic FTMH with cystoid edema. (**r, s**) GAN-based GDLMs predict clear postoperative OCT images. (**t**) However, CycleGAN does not modify the spatial details of the input OCT. (**u**) In the GT-OCT image, a foveal notch appears after hole closure, along with EZ disruption. (**v, w**) VAE models simulate the innermost retinal boundary similar to the GT-OCT image. (**x**) Our proposed model predicts the discontinuation of the ELM and EZ, accompanied by high intensity due to glial proliferation around the fovea.

Supplementary Figure S8. Bland-Altman plot results between thickness parameters reflecting marcular molphology.

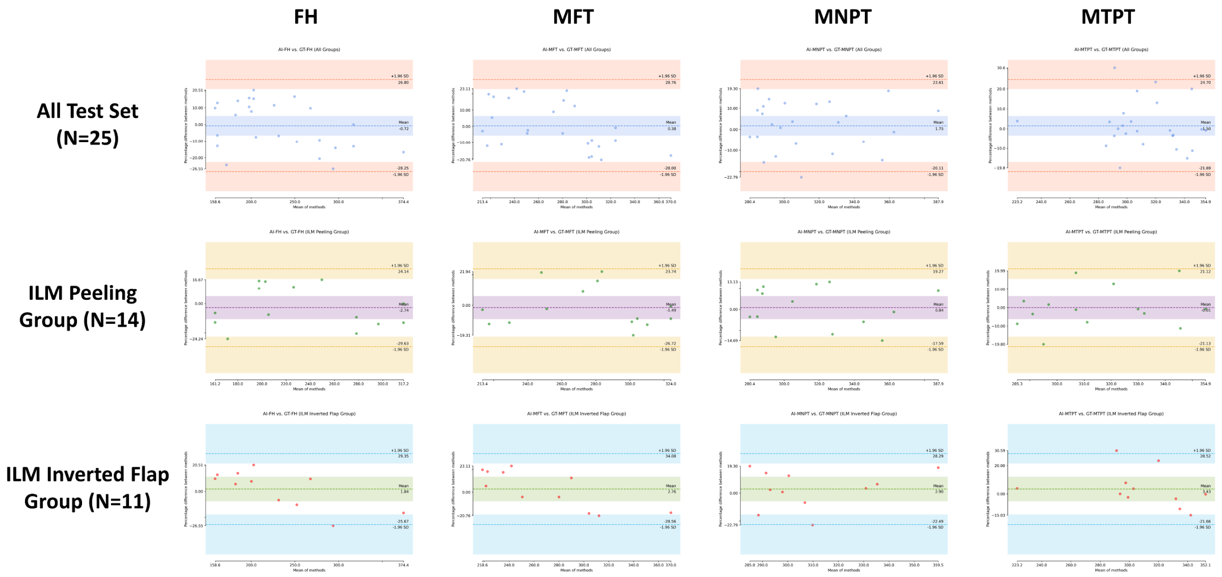

Bland-Altman plots were presented for all test sets, the internal limiting membrane (ILM) peeling group, and the ILM inverted flap group, arranged from the top to the bottom. Each column from left to right represents Bland-Altman plots for foveolar height (FH), mean foveal thickness (MFT), mean nasal parafoveal thickness (MNPT), and mean temporal parafoveal thickness (MTPT). These retinal thickness profiles from AI-OCT were presented on the y-axis as the bias and the upper and lower bounds of the 95% limits of agreement (LoA), expressed as a percentage difference in reference to the profiles measured in GT-OCT. In all test sets and the ILM peeling group, the 95% LoA for all parameters fell within the 30% range of the cut-off value for LoA. However, for the ILM inverted flap group, the upper 95% LoA for MFT exceeded 30%. As a result, GDLM successfully predicted macular morphology in compliance with the cut-off value's LoA, except for MFH in the ILM inverted flap group.

Supplementary Table S9. Logistic regression analysis of external limiting membrane and ellipsoid zone disruption with baseline and intraoperative factors

Factors for ELM Disruption	Univariate Logistic Regression		Multivariate Logistic Regression[a]	
	**Odds Ratio (95% CI)**	***P*-value**	**Odds Ratio (95% CI)**	***P*-value**
Preoperative CSMT (μm)	0.994 (0.989-0.998)	0.009	-	-
Hole size (μm)	1.006 (1.003-1.008)	0.022	1.006 (1.003-1.008)	< 0.001
FTMH Stage (2→4)	2.032 (1.255-3.288)	0.004	-	-
Combined surgery	0.393 (0.177-0.873)	0.022	-	-
ILM manipulation technique (Inverted flap)	4.617 (2.008-10.617)	< 0.001	-	-

Factors for EZ Discontinuation	Univariate Logistic Regression		Multivariate Logistic Regression[b]	
	**Odds Ratio (95% CI)**	***P*-value**	**Odds Ratio (95% CI)**	***P*-value**
Preoperative CSMT (μm)	0.995 (0.990-0.999)	0.017	-	-
Hole size (μm)	1.005 (1.003-1.007)	< 0.001	1.004 (1.002-1.006)	< 0.001
Combined surgery	0.436 (0.202-0.943)	0.035	-	-
ILM manipulation technique (Inverted flap)	7.368 (3.221-16.858)	< 0.001	4.254 (1.746-10.363)	0.001

CI = confidence interval, CSMT = central subfield mean thickness, ELM = external limiting membrane, EZ = ellipsoid zone, FTMH = full-thickness macular hole, ILM = internal limiting membrane.

[a] = Preoperative CSMT, Hole size, CSMT, higher FTMH Stage, Combined surgery, and ILM inverted flap technique were tested as independent variables for the multivariate logistic regression analysis.

[b] = Preoperative CSMT, Hole size, CSMT, Combined surgery, and ILM inverted flap technique were tested as independent variables for the multivariate logistic regression analysis.

Supplementary Figure S10. Confusion matrices for prediction of external limiting membrane and ellipsoid zone restoration.

All Test Set (N=25)

ELM Restoration (N = 25)		GT-OCT	
		Restoration	Disruption
AI-OCT	Restoration	**17**	**2**
	Disruption	**1**	**5**

Accuracy = 88.0%, F1 score = 0.919
Impurity = 0.190

EZ Recovery (N = 25)		GT-OCT	
		Success	Failure
AI-OCT	Success	**15**	**2**
	Failure	**0**	**8**

Accuracy = 92.0%, F1 score = 0.938
Impurity = 0.128

ILM Peeling Group (N=14)

ELM Restoration (N = 14)		GT-OCT	
		Restoration	Disruption
AI-OCT	Restoration	**12**	**1**
	Disruption	**1**	**0**

Accuracy = 85.7%, F1 score = 0.923
Impurity = 0.132

EZ Recovery (N = 14)		GT-OCT	
		Success	Failure
AI-OCT	Success	**11**	**1**
	Failure	**0**	**2**

Accuracy = 92.9%, F1 score = 0.957
Impurity = 0.095

ILM Inverted Flap Group (N=11)

ELM Restoration (N = 11)		GT-OCT	
		Restoration	Disruption
AI-OCT	Restoration	**5**	**1**
	Disruption	**0**	**5**

Accuracy = 90.9%, F1 score = 0.909
Impurity = 0.152

EZ Recovery (N = 11)		GT-OCT	
		Success	Failure
AI-OCT	Success	**4**	**1**
	Failure	**0**	**6**

Accuracy = 90.9%, F1 score = 0.889
Impurity = 0.156

The results of predicting the restoration of the external limiting membrane (ELM, upper row) and recovery of the ellipsoid zone (EZ, lower row) using the test set are illustrated in a confusion matrix for each group. Each group's accuracy and F1 score are recorded in the bottom left corner of the matrix. We calculated the GINI impurity for each confusion matrix and included the impurity values in the lower left corner. The accuracy and F1 score for all groups (left column) show favorable predictions with values equal to or exceeding 88.0% and 0.889, respectively. The recall rates for ELM restoration and disruption in the entire group are 94.4% (17/18) and 71.4% (5/7), respectively, in all groups. In inverted internal limiting membrane flap groups, the recall rate for the success of EZ recovery is high at 100.0% (4/4), whereas for the failure of EZ recovery, it is at 85.7% (6/7). The impurity values for ELM and EZ restoration were 0.190 and 0.128, respectively.

Supplementary Figure S11. Prediction of postoperative optical coherence tomography images with varying conditional vectors.

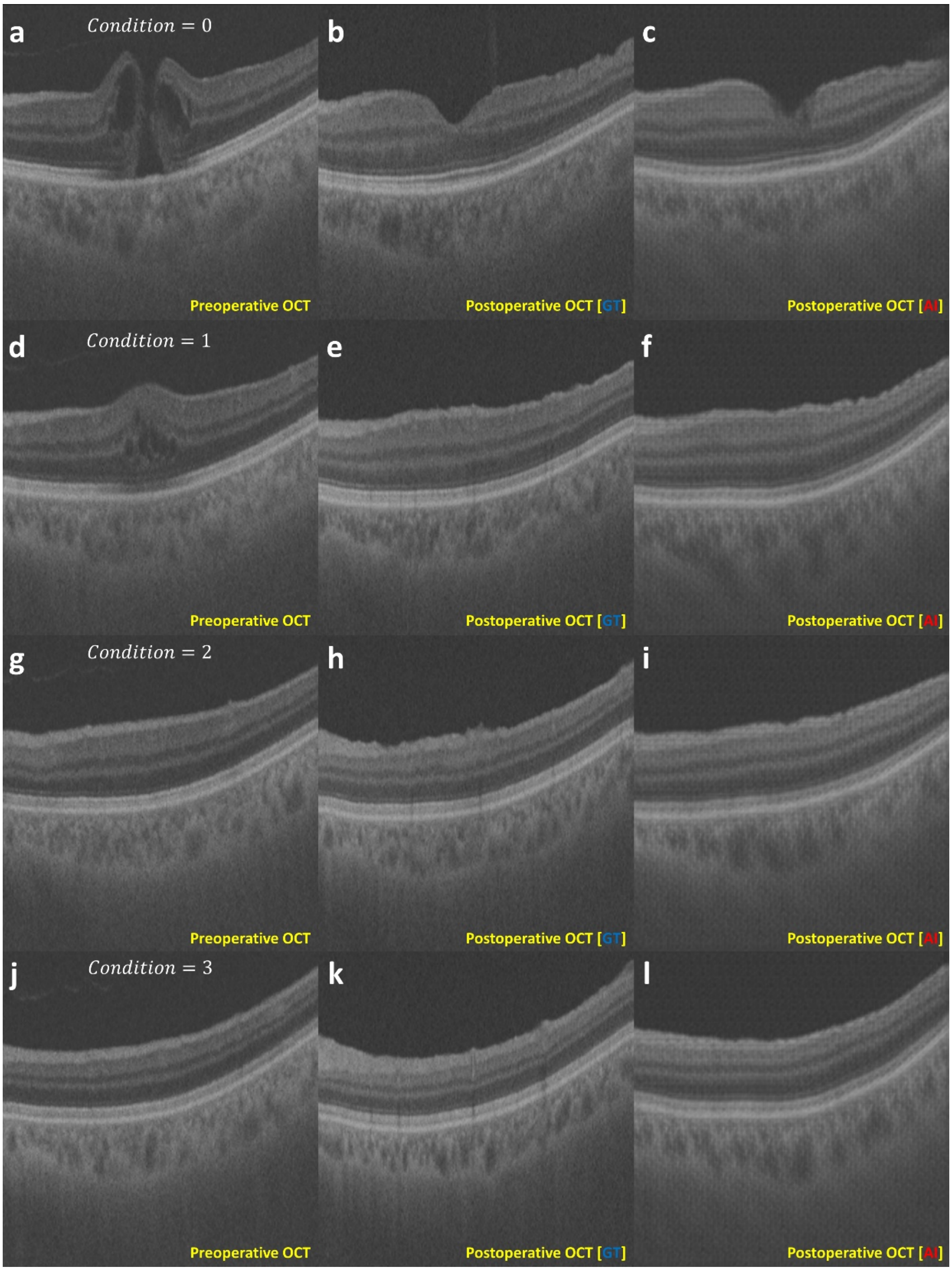

The left column shows the preoperative optical coherence tomography (OCT) images of the test set. The conditions range from 0 to 3 based on their distances from the fovea. The middle column shows the postoperative ground truth (GT) OCT (GT-OCT) images, whereas the rightmost column shows the postoperative predicted artificial intelligence (AI) OCT (AI-OCT) images. (**a**–**c**) The condition vector is assigned to 0, corresponding to the OCT cross-sections passing through the fovea. The foveal contours in the (**b**) GT- and (**c**) AI-OCT images are remarkably similar. (**d**) The cross-sectional OCT passes through 30 slices (703 μm) away from the foveola, and the condition vector is assigned to 1. The (**e**) GT- and (**f**) AI-OCT images are similar, and foveal depression is no longer visible. (**g**–**i**) The OCT images are situated at 60 slices (1406 μm) away from the foveola, and the condition vector is now set to 2. All layers of the retina are well delineated in the AI-OCT images (**i**). (**j**–**l**) The OCT images at 90 slices (2110 μm) are illustrated, and the condition vector is assigned to 3. The AI-OCT image (**l**) is almost identical to the retina depicted in the GT-OCT image (**k**).
